# A systematic review of methods to diagnose oral dryness and salivary gland function

**DOI:** 10.1186/1472-6831-12-29

**Published:** 2012-08-08

**Authors:** Christina Diogo Löfgren, Claes Wickström, Mikael Sonesson, Pablo Tapia Lagunas, Cecilia Christersson

**Affiliations:** 1 Department of Materials Science and Technology, Faculty of Odontology, Malmö University, Malmö, Sweden; 2 Department of Oral Biology, Faculty of Odontology, Malmö University, Malmö, Sweden; 3 Department of Orthodontics, Faculty of Odontology, Malmö University, Malmö, Sweden; 4 Library and IT Services, Digital Information Services, Malmö University, Malmö, Sweden; 5 Department of Materials Science and Technology, Faculty of Odontology, Malmö University, Malmö, Sweden

## Abstract

**Background:**

The most advocated clinical method for diagnosing salivary dysfunction is to quantitate unstimulated and stimulated whole saliva (sialometry). Since there is an expected and wide variation in salivary flow rates among individuals, the assessment of dysfunction can be difficult. The aim of this systematic review is to evaluate the quality of the evidence for the efficacy of diagnostic methods used to identify oral dryness.

**Methods:**

A literature search, with specific indexing terms and a hand search, was conducted for publications that described a method to diagnose oral dryness. The electronic databases of PubMed, Cochrane Library, and Web of Science were used as data sources. Four reviewers selected publications on the basis of predetermined inclusion and exclusion criteria. Data were extracted from the selected publications using a protocol. Original studies were interpreted with the aid of Quality Assessment of Diagnostic Accuracy Studies (QUADAS) tool.

**Results:**

The database searches resulted in 224 titles and abstracts. Of these abstracts, 80 publications were judged to meet the inclusion criteria and read in full. A total of 18 original studies were judged relevant and interpreted for this review. In all studies, the results of the test method were compared to those of a reference method.

Based on the interpretation (with the aid of the QUADAS tool) it can be reported that the patient selection criteria were not clearly described and the test or reference methods were not described in sufficient detail for it to be reproduced. None of the included studies reported information on uninterpretable/intermediate results nor data on observer or instrument variation. Seven of the studies presented their results as a percentage of correct diagnoses.

**Conclusions:**

The evidence for the efficacy of clinical methods to assess oral dryness is sparse and it can be stated that improved standards for the reporting of diagnostic accuracy are needed in order to assure the methodological quality of studies. There is need for effective diagnostic criteria and functional tests in order to detect those individuals with oral dryness who may require oral treatment, such as alleviation of discomfort and/or prevention of diseases.

## Background

Oral dryness is a complex condition, expressed as a physiological deficiency with or without perceived dysfunction. Clinically, oral dryness may vary from a slight reduction in salivary flow with transient inconvenience to severe impairment of oral health and concomitant psychological indisposition. Salivary dysfunction has mainly been related to a decrease in salivary flow rate, but the molecular composition of saliva has gained more attention in understanding the complexity of the condition. Saliva has been shown to have multi- functional characteristics as expressed by several families of salivary molecules, each comprising multiple members that are multifunctional and overlapping [1]. This explains the presence of a compensatory mechanism in saliva and that the expression of salivary dysfunction is most likely to be multi-facetted.

The prevalence of oral dryness reported in the literature varies from 10% to 80% [2-12].

Part of this variation might be explained by the fact that there is no global consensus regarding the terminology associated with oral dryness, although many authors distinguish between xerostomia, denoting the subjective feeling and hyposalivation, denoting a decreased salivary flow rate [13-17].

Oral dryness may be caused by many factors. One factor of importance is pharmacotherapy. Decreased salivary flow rate has been reported as a consequence of treatment with various types of drugs but as for the subjective feeling of oral dryness the total number of drugs taken seems to be more important [18]. No age-dependent decrease in salivary flow rate has been established [19,20] although a higher prevalence of perceived oral dryness has been reported with increased age [18]. This might be explained by an increased intake of medication with increasing age.

Several systemic disorders such as Sjögren’s syndrome, diabetes mellitus, rheumatoid arthritis, and systemic lupus erythematosus are also known to cause oral dryness. In addition, radiotherapy to the head and neck region is a factor of importance.

With regard to an increasingly elderly population and its dependent use of medication, a multitude of negative side effects associated with disturbed salivary function will present a medico-socio-economic problem not only for the individual *per se* but also for society in the near future.

The most advocated clinical method for diagnosing salivary gland dysfunction is to quantitate unstimulated and stimulated whole saliva flow rates (sialometry). The cut-off value for a very low unstimulated and stimulated whole saliva flow rate is claimed to be ≤ 0.1 ml/min and ≤ 0.7 ml/min, respectively which is generally referred in the literature to studies by Ericsson and Hardwick [21] and Sreebny and Valdini [4]. Attempts have also been made to correlate perceived oral dryness to salivary output with varying results. However, reports where the patients describe their oral comfort/discomfort levels, suggest weak to no correlation between measures of salivary flow rates and an individual’s own subjective description [4,22-24]. Symptoms of dry mouth often occur when the salivary flow rate is reduced by about 50%, but can also be experienced within what is regarded as the normal salivary flow rate range [25]. Unless the mouth is almost dry, without proper individual baseline information it is almost impossible to ascertain if the level of a patient’s salivary flow rate is actually below ‘normal’. Since there is a great variability in individual salivary flow rate and a wide range of flow rate is accepted, the accurate assessment of dysfunction can be difficult. With this in mind, it can be argued if measurements of salivary flow rates can be used as a discriminating diagnostic tool.

Ghezzi et al. [26] studied the variation of stimulated parotid and submandibular flow rates in a healthy population of adults over a six-hour period. In accordance with other investigators [25,27], they demonstrated that salivary flow rates are not constant and that there exists a broad range of stimulated parotid and submandibular flow rates among healthy individuals. These findings were confirmed in the study by Burlage et al. [28]. They determined the variation in stimulated parotid flow rate for repeated collections. They also found a large degree of similarity between the flow rates of the left and right parotid glands in healthy subjects as well as in patients with Sjögren’s syndrome.

Responses to standardized questions have been compared to objective measures of major salivary gland output in order to identify certain questions that with a high degree of reliability predict true salivary gland dysfunction [22]. The results showed that questions focusing on perceived oral dryness associated with eating were highly indicative of salivary performance whilst the most commonly heard complaints such as perceived oral dryness at night and during the day had no significant correlation with reduced salivary flow rate.

Furthermore, the individuals’ assessment of the quantity of saliva as “too little” was highly predictive of decreased salivary output.

The aim of this systematic review is to evaluate the quality of the evidence for the efficacy of diagnostic methods used to identify oral dryness.

## Methods

The systematic approach of the literature review was adapted according to Goodman [29] and consisted of the following steps: specification of the problem, formulation of a plan to conduct the literature search with specified indexing terms, retrieval of publications and interpretation of the evidence from the literature studied.

### Specification of the problem

The following question was developed to articulate the problem: What methods are used to diagnose oral dryness and what is the efficacy of these methods?

### Formulation of a plan, literature search, and retrieval

The first step of the search was to use Medical Subject Headings (MeSH) and free text words to search the electronic databases PubMed (including MeSH Subheadings and [All fields]), Cochrane Library, and Web of Science.”

The following search terms were identified on the basis of Medical Subject Headings (MeSH) and within the MeSH database these terms are defined as:

• Saliva: the clear, viscous fluid secreted by the salivary glands and mucous glands of the mouth and containing mucins, water, organic salts, and ptylin.

• Xerostomia: decreased salivary flow.

• Diagnostic techniques and procedures: methods, procedures, and tests performed to diagnose disease, disordered function, or disability. Year introduced: 1998.

• Pilocarpine: a slowly hydrolyzed muscarinic agonist with no nicotinic effects. Pilocarpine is used as a miotic and in the treatment of glaucoma.

The above mentioned MeSH terms were combined by using the Boolean operator ‘OR’ with free text words within a search facet.

Since this review focused on diagnostic methods and an initial search resulted in a number of publications on intervention with pilocarpine, the decision was made to confine the search by excluding those studies.

Exclusion of studies on intervention with pilocarpine was obtained by using the Boolean operator ‘NOT’ in the search.

To be included in this review, publications that described a method for diagnosing oral dryness were searched. The inclusion and exclusion criteria are presented in Table 1.

**Table 1:** Inclusion and exclusion criteria

**Inclusion criteria**	**Exclusion criteria**
Human studies	Review articles and abstracts
Oral dryness primary condition	Studies on pharmacologic intervention with sialogogues (e.g. pilocarpine)
Study population presenting symptoms and/or findings of oral dryness	Studies on the effect of radiation therapy No control group
Standardized conditions for donors	Studies where oral dryness is a secondary outcome variable
Articles written in English	

Inclusion and exclusion criteria for the retrieved studies.

The search was limited to publications with an Entrez date in the period from January 1, 1966 to February 22, 2011.

The decision to include the article was made by reading the title and the abstract. Four authors (CDL, CW, MS and CC) screened all titles and abstracts independently for possible inclusion. When an abstract was considered by at least one author to be relevant, the full text was obtained for independent assessment against the stated inclusion criteria (Table 1) with the aid of a protocol (Additional file 1: Table S1). Any disagreement was resolved by discussion among the reviewers.

The protocol used in the assessment against the stated inclusion criteria.

Will the study be included in the systematic review? Yes No.

The second step was to search by hand the reference lists of the original studies that had been found relevant in the first step. Reference lists of reviews were also searched. Titles containing words suggesting diagnostic measures and techniques of oral dryness were sought.

Inclusion and exclusion criteria were also considered in the hand search. The abstracts of the selected references were obtained and when considered relevant by at least one author, the publication was ordered in full text. Book chapters and reviews were excluded, since the search focused on original studies.

### Data extraction

Data were extracted on the following items: year of publication; study objectives; study design; study population; control group; test method; reference method; and authors’ conclusion.

Original studies that presented a method for diagnosing oral dryness were interpreted according to the Quality Assessment of Diagnostic Accuracy Studies (QUADAS) tool [30].

In the present review, a modified protocol comprising 15 questions was applied, as presented in Table 2.

**Table 2:** Protocol based on the Quadas tool for interpretation of relevant original studies

		**Yes**	**No**	**Unclear**
1	Was the spectrum of patients representative of the patients who will receive the test in practice?	()	()	()
2	Were the selection criteria clearly described?	()	()	()
3	Is there a diagnostic reference standard?	()	()	()
4	If so, is the reference standard likely to correctly classify the target condition?	()	()	()
5	Is the time period between reference standard and index test short enough to be reasonably sure that the target condition did not change between the two tests?	()	()	()
6	Did the whole sample or a random selection of sample, receive verification using a reference standard of diagnosis?	()	()	()
7	Was the execution of the index test described in sufficient detail to permit replication of the test?	()	()	()
8	Was the execution of the reference standard described in sufficient detail to permit replication of the test?	()	()	()
9	Were the index test results interpreted without knowledge of the results of the reference standard?	()	()	()
10	Were the reference standard results interpreted without knowledge of the results of the index test?	()	()	()
11	Were the same clinical data available when test results were interpreted as would be available when the test is used in practice?	()	()	()
12	Were uninterpretable/intermediate results reported?	()	()	()
13	Were withdrawals from the study explained?	()	()	()
14	Are data presented on observer or instrument variation that could affect the estimates of test performance?	()	()	()
15	Were appropriate results presented (percentage of correct diagnoses, sensitivity, specificity, predictive values, measures of ROC, likelihood ratios, or other relevant measures) and were these calculated appropriately?	()	()	()

Interpreter:

Date:

The same reviewers assessed the articles separately. Any interexaminer disagreements were resolved by discussion on each article to reach a consensus.

## Results

**Figure 1:**
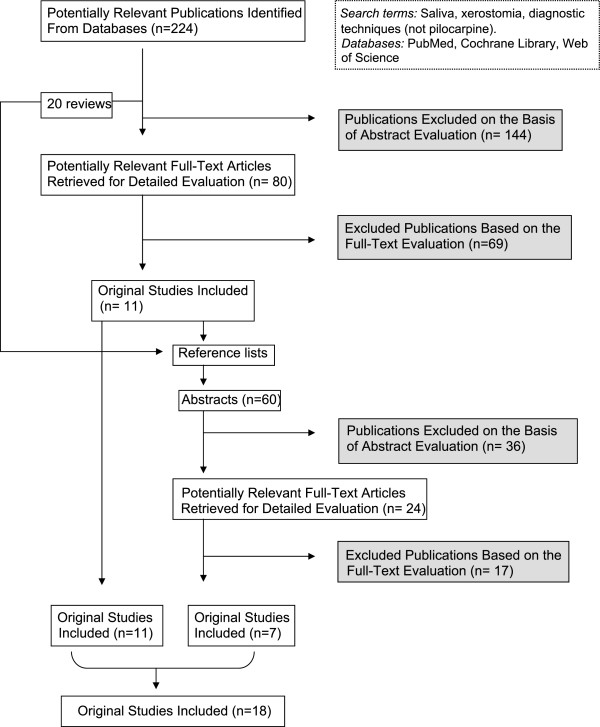
**Flow chart. **Flow chart and selection process of the included publications.

The number of publications retrieved, read and interpreted is presented in Figure 1.

The database searches resulted in 224 titles and abstracts. Of these abstracts, 80 publications were judged to meet the inclusion criteria and read in full. Data were extracted according to the pre-established protocol. Animal studies and publications in a language other than English were excluded. Publications using a sialogogue (not only pilocarpine) as intervention were excluded. Furthermore, studies where oral dryness was not described as a primary condition, studies where the conditions for the donors were not standardised and studies that did not include a control group were excluded. Studies were oral dryness was a secondary outcome variable, i.e. studies reporting oral dryness as a secondary finding to e.g. treatment or medication, but not to general diseases e.g. Sjögren’s syndrome, were also excluded.

This resulted in 11 original studies being considered relevant for the review. The hand search of the reference lists of 11 original studies and 20 reviews resulted in an additional 60 abstracts. After these abstracts had been read, 24 publications were selected and read in full and data were extracted. After the publications had been read in detail, seven were selected.

A total of 18 original studies were interpreted for this review [4,31-47].

Preliminary evaluation of the included studies revealed heterogeneity with respect to inclusion criteria, test method and reference method. Consequently, it was not possible to conduct a quantitative data synthesis leading to a meta-analysis.

### Study objectives and study design

Seven of the included studies [31,33,34,36,37,43,44] aimed at examining the potential use of different tests or a combination of tests for the diagnosis of Sjögren’s syndrome. The remaining 11 studies [4,32,35,38-42,45-47] described different methods for determining salivary hypofunction.

Sixteen of the studies [31-34,36-47] were case control studies, and two [4,35] were cross sectional studies.

Five studies were performed in the USA [4,31,39,42,46], two in The Netherlands [36,37], two in Sweden [32,34], one in Egypt [33], one in Great Britain [35], one in Japan [38], one in South Korea [40], one in Spain [41], one in Denmark [43], one in France [44], one in Mexico [45] and one in Israel [47].

### Study populations and control groups

The majority of de individuals included in the study groups were individuals with Sjögren’s syndrome (Table 3). In six studies [4,38,40,42,46,47] the study population consisted of individuals with symptoms of and/or findings of oral dryness. In one study [35], subjects were participants in a cross sectional general health survey, randomly selected from a population register.

**Table 3:** Data of study populations and control groups in the studies included in the review

**First author, year of publication**	**Study population**	**Control group**
Al-Hashimi 1998 [31]	43 subjects**:**	39 age/gender/race matched healthy control subjects
	−38 with pSS^a^	
	−5 with sSS^b^	
	Age and gender not stated	
Almståhl 2003 [32]	39 subjects:	12 control subjects
	−9 subjects with history of head and neck radiation	(10 females/2 males) with normal salivary secretion rates *Mean age 54 yrs*
	(2 females/8 males^c^)	
	*Mean age 55 yrs*	
	−10 subjects with pSS	
	(10 females)	
	*Mean age 57 yrs*	
	−10 subjects treated with neuroleptic injections	
	(4 females/6 males)	
	*Mean age 44 yrs*	
	–10 subjects with hyposalivation due to medication or unknown origin	
	(9 females/1 male)	
	*Mean age 54 yrs*	
El-Miedany 1999 [33]	25 subjects:	15 healthy age-matched controls
	−15 with pSS	*Mean age 50 yrs*
	−10 with sSS	10 younger controls
	(20 females/5 males)	*Mean age 26.2 yrs*
	Mean age 48 yrs	Gender not stated
Håkansson 1994 [34]	17 subjects with pSS (15 females/2 males)	30 subjects with symptoms of dry mouth and/or eyes
	*Mean age 63 yrs*	(21 females/9 males)
		*Mean age 69 yrs*
		12 subjects without symptoms of dry mouth and/or eyes
		(6 females/6 males)
		*Mean age 68 yrs*
Hay 1998 [35]	341 subjects:	
	−189 females	
	*Age (median) 49 yrs*	
	−152 males	
	*Age (median) 46 yrs*	
First author, year of publication	Study population	Control group
Kalk 2001 [36]	100 consecutive subjects:	36 healthy subjects
	−33 with pSS	(20 females/16 males)
	(30 females/3 males)	*Mean age 39 yrs*
	*Mean age 51 yrs*	
	−25 with sSS	
	(21 females/4 males)	
	*Mean age 54 yrs*	
	−42 tested negative for SS	
	(40 females/2 males)	
	*Mean age 55 yrs*	
Kalk 2002 [37]	20 subjects:	100 subjects (from previous study reported above-see study population)
	−2 with pSS	
	−5 with sSS	
	(6 females/1 male)	
	*Mean age 62 yrs*	
	−13 non-SS	
	(13 females)	
	*Mean age 55 yrs*	
Kanehira 2009 [38]	9 subjects with complaints of decreased salivary flow rate	31 healthy subjects
	Age and gender reported for all included subjects	
	(22 females/19 males)	
	*Mean age 48.8 yrs*	
Kohn 1992 [39]	22 subjects:	33 healthy volunteers
	−19 with pSS	(20 females/13 males)
	−1 with sSS	*Mean age 54.4 yrs*
	−2 with idiopathic xerostomia	
	(17 females/5 males)	
	*Mean age 52.2 yrs*	
Lee 2002 [40]	20 subjects with dry mouth	20 age/gender matched healthy control subjects
	(unstimulated whole saliva flow rate <0.15 ml/min)	(18 females/2 males)
	(18 females/2 males)	*Mean age 42.6 yrs*
	*Mean age 43.6 yrs*	
First author, year of publication	Study population	Control group
López-Jornet 2006 [41]	92 subjects:	70 healthy volunteers
	−61 with pSS or sSS	(35 females/35 males)
	(50 females/11 males)	*Mean age 40.53 yrs*
	*Mean age 57.08 yrs*	
	−31 displaying symptoms of oral dryness from different causes	
	(29 females/2 males)	
	*Mean age 52.52 yrs*	
Navazesh 1992 [42]	A total of 71 subjects were included.	Unclear
	Approx. half with salivary gland hypofunction, defined as unstimulated whole salivary flow rate ≤0.20 ml/min	Approx. half of the total number of included subjects. Individuals with normal salivary flow rates.
	(48 females/23 males)	
	*Mean age 52 yrs*	
Pedersen 1999 [43]	16 subjects:	14 healthy volunteers
	(14 females/2 males) meeting the 1993 European classification for pSS.	(13 females/1 male)
	*Mean age unclear*	*Mean age 50 yrs*
		13 healthy volunteers
		(12 females/1 male)
		*Mean age 24 yrs*
Pennec 1993 [44]	72 consecutive subjects:	14 healthy subjects
	- 40 subjects with pSS	*Mean age 64.7 yrs*
	−16 subjects with sSS	Gender not stated
	−16 subjects with connective tissue disease but no evidence of sSS	
	(70 females/2males)	
	*Mean age 59.4 yrs*	
Sánchez-Guerrero 2002 [45]	90 subjects:	152 healthy subjects
	−30 subjects with pSS	(106 females/46 males)
	(29 females/1 male)	*Mean age 35.2 yrs*
	*Mean age 50.6 yrs*	
	−60 subjects with CTDs without SS	
	(50 females/10 males)	
	*Mean age 37.4 yrs*	
First author, year of publication	Study population	Control group
Sreebny 1988 [4]	151 subjects with reported dry mouth	372 subjects without reported dry mouth
	(109 females/42 males)	(219 females/159 males)
	*Age range 18–55+*	*Age range 18–55+*
Wolff 1998 [46]	50 subjects:	25 subjects with no perception of dry mouth and no medication with resting salivary flow rates > 1.0 ml/min
	−25 subjects with complaints of ‘dry mouth’ due to medication, with resting salivary flow rates ≤0.1 ml/min	(15 females/10 males) *Mean age 44*
	(20 females/5 males)	
	*Mean age 48.5*	
	−25 subjects with complaints of ‘dry mouth’ due to medication, with resting salivary flow rates > 0.1 ml/min	
	(18/females/7 males)	
	*Mean age 49.2*	
Wolff 2002 [47]	27 subjects with complaints of dry mouth	32 healthy subjects
	Age and gender not stated	Age and gender not stated

^a ^pSS = primary Sjögren’s syndrome.

^b ^sSS = secondary Sjögren’s syndrome.

^c ^Female/male ratio according to table in original study.

The control groups consisted mainly of age- and gender-matched healthy individuals. In two studies, the control group consisted of individuals with normal salivary secretion rates [32,42]. One study [34] included individuals with symptoms of dry mouth in the control group, and in one study [37] the control group consisted of individuals with Sjögren’s syndrome.

The number of included individuals in the study populations varied between 16 and 341 and in the control groups between 12 and 372.

Two studies did not report age and gender [31,47] for individuals included in the study populations or control groups. Two studies [33,44] did not state gender for individuals included in the control groups. Two studies [38,42] reported age and gender for all included subjects.

The female/male ratio in the study populations 783/273 [4,32-37,39-41,43-45,47] and control groups were and 586/309 [4,32,34,36,37,39-41,43,45,46], respectively.

### Index test methods and reference methods

A variety of index test methods were used in the included articles (Table 4). .

**Table 4:** Data of index test methods and reference methods in the studies included in the review

**First author, year of publication**	**Test method**	**Reference method**	**Authors’ conclusion**
Al-Hashimi 1998 [31]	3 different gel electrophoresis systems	European Community criteria for the diagnosis of Sjögren’s syndrome [42]	Salivary electrophoresis is a potentially useful test for the diagnosing of Sjögren’s syndrome
	▪SDS-PAGE		
	▪anionic-PAGE		
	▪immobilized pH gradient (IPG)		
Almståhl 2003 [32]	Sialochemistry	The Copenhagen criteria for Sjögren’s syndrome [47]	The concentrations of electrolytes in stimulated whole saliva, in individuals with hyposalivation, seem to be more related to the reason for the hyposalivation than to the salivary secretion rate.
El-Miedany 1999 [33]	Salivary smears	Criteria of Fox et al. for Sjögren’s syndrome [48]	The saliva ferning test is a useful diagnostic aid in autoimmune xerostomia, approx equivalent to Shrimer’s test in xeropthalmia
Håkansson 1994 [34]	^99m^Tc-scintigraphy	▪Copenhagen criteria for pSS [47]	Salivary gland scintigraphy is a sensitive and valid method to measure salivary gland function and abnormalities
		▪Shrimer-1 test	
		▪van Bijsterveld score	
		▪Tear-film break-up time	
Hay 1998 [35]	Questionnaire	▪Sialometry [42-44]	Only weak associations were found between self-reported symptoms of dry eyes and dry mouth and objective measures said to define Sjögren’s syndrome in the general population
		▪Shrimer-1 test	
		▪Measurement of antibodies (ELISA, indirect immunofluorescence, Latex test)	
Kalk 2001 [36]	▪Sialometry	Revised European classification criteria for SS [43-45]	Glandular sialometry and sialochemistry are not useful tools for differentiating SS from other salivary gland disease.
	▪Sialochemistry	Parotid sialography was used to fulfill the criteria on the oral component.	
Kalk 2002 [37]	▪Sialometry	Revised European classification criteria for SS [43-45]	Gland-specific sialometry and sialochemistry may eventually replace other, more invasive, diagnostic techniques for diagnosing SS.
	▪Sialochemistry	Parotid sialography was used to fulfill the criteria on the oral component.	
Kanehira 2009 [38]	Filter paper comprising 3 spots containing 30 μg starch and 49.6 μg potassium iodide per spot.	Sialometry (unstimulated whole saliva < 0.1 ml/min)^a^	This screening technique might be effective for estimation of salivary flow.
Kohn 1992 [39]	^99m^Tc-scintigraphy	Sialometry (Unstimulated parotid and SM/SL < 0.1 ml/min Stimulated parotid and SM/SL < 0.6 ml/min)*	Salivary gland scintigraphy is a useful method in assessing salivary gland flow rates
Lee 2002 [40]	▪Oral mucosal wetness	Sialometry (unstimulated whole saliva <0.15 ml/min) ^a^	Measurements of oral mucosal wetness could be thought of as one of the diagnostic methods for assessing dry mouth.
	▪Sialochemistry		
	(Sialopaper^TM^ Periotron 8000®)		
			
López-Jornet 2006 [41]	Oral Schirmer’s test	Revised European classification criteria for SS [46]	Oral Shrimer’s test can be used as a simple, objective test to diagnose salivary gland hypofunction.
Navazesh 1992 [42]	▪Lip dryness	Sialometry (unstimulated whole saliva ≤0.20 ml/min) ^a^	Four clinical measures that together predict the presence of or absence of salivary gland hypofunction were identified: dryness of lips, dryness of buccal mucosa, absence of saliva produced by gland palpation, and total DMFT.
	▪Buccal mucosal dryness		
	▪Salivary pool		
	▪Major salivary gland palpation		
	▪Tongue mucosa		
	▪Periodontium		
	▪Total DMFT		
Pedersen 1999 [43]	▪Sialometry	European classification for pSS [42]	Rating of oral dryness by visual analogue scales or categorised questionnaires are valuable for the evaluation of oral involvement in pSS.
	▪Labial salivary gland biopsy		
	▪Serological examination		
	▪Interview		
	▪Categorised questionnaire		
	▪VAS		
Pennec 1993 [44]	▪Sialometry	European classification for pSS [42]	The most efficient combination of tests for the oral component of SS appears to be salivary gland scintigraphy plus saliva flow rate or salivary lactoferrin.
	▪Salivary lysozyme		
	▪Salivary lactofferin		
	▪Parotic sialography		
	▪Salivary gland scintigraphy (^99m^Tc)		
	▪Labial salivary gland biopsy		
Sánchez-Guerrero 2002 [45]	Wafer test	For the oral component parotid secretion rate according to Fox et al. [48]	The wafer test is valid and reliable for identifying subjects with xerostomia
		▪European questionnaire for sicca syndrome	
		▪Schirmer-1 test	
Sreebny 1988 [4]	Questionnaire	Sialometry (unstimulated whole saliva ≤ 0.1 ml/min) ^a^	Dry mouth and several other symptoms are common in an outpatient population and they are a valid indicator of salivary gland hypofunction
Wolff 1998 [46]	Oral mucosal wetness(Sialopaper^TM^ Periotron 6000®)	Sialometry (unstimulated whole saliva ≤ 0.1 ml/min) ^a^ Salivary pH	Measurement of palatal mucosal wetness might be useful in assessing medication compliance and may serve as a guide to medication administration.
Wolff 2002 [47]	3-g-all-sucrose candy	Sialometry (unstimulated SM/SL < 0.1 ml/min + either stimulated parotid <0.25 ml/min or stimulated SM/SL < 0.15 ml/min) ^a^	The candy weight-loss test is a measure of salivary hypofunction, which correlates with saliva output and reports of subjective dry mouth

^a ^Cut-off value defining salivary gland hypofunction

In general terms, the index tests could be divided into five categories:

(1) Secretion tests; including sialometry and sialochemistry,Oral Schirmer’s test, secretion composition using protein separation techniques, total protein content, electrolyte content and specific protein content with immunological tests or iodine starch reactions (amylase) [31,32,36-38,41]

(2) Mucosal/surface tests; including mucosal dryness/residual wetness tests, salivary smears and biopsies [33,46]

(3) ‘Functional’ tests; including dissolutions tests of candy or wafers [45,47]

(4) Glandular morphology; including scintigraphy or sialography [31,36]

(5) Questionnaires and/or interviews [4,34].

Some studies used a combination of tests to assess salivary gland hypofunction [40,42-44]. All studies used sialometry as a reference standard when evaluating the different index tests. However, different secretions, including stimulated and unstimulated glandular saliva, as well as unstimulated whole saliva were used (Table 5).

**Table 5:** Salivary secretions used as reference method in the included studies

**First author, year of publication**	**Secretion**
Al-Hashimi 1998 [31]	Stimulated parotid saliva (2%citric acid)
Almståhl 2003 [32]	Unstimulated whole saliva
El-Miedany 1999 [33]	Unstimulated whole saliva
Håkansson 1994 [34]	Unstimulated whole saliva
Hay 1998 [35]	Unstimulated whole saliva
Kalk 2001 [36]	Unstimulated and stimulated (2% citric acid) parotid and SM/SL saliva
Kalk 2002 [37]	Unstimulated and stimulated (2% citric acid)parotid and SM/SL saliva
Kanehira 2009 [38]	Unstimulated whole saliva
Kohn 1992 [39]	Unstimulated and stimulated (2% citric acid) parotid and SM/SL saliva
Lee 2002 [40]	Unstimulated whole saliva
López-Jornet 2006 [41]	Unstimulated and stimulated (4% citric acid) whole saliva
Navazesh 1992 [42]	Unstimulated and stimulated (inert gum base) whole saliva and stimulated (candy-stimulated and acid-stimulated 0.15 mol/L citric acid) parotid saliva.
Pedersen 1999 [43]	Unstimulated whole saliva
Pennec 1993 [44]	Unstimulated whole saliva
Sánchez-Guerrero 2002 [45]	Unstimulated whole saliva
Sreebny 1988 [4]	Unstimulated and stimulated (paraffin) whole saliva
Wolff 1998 [46]	Unstimulated whole saliva
Wolff 2002 [47]	Unstimulated and stimulated (2% citric acid) parotid and SM/SL saliva

Eleven of the included studies [31-37,41,43-45] used established criteria for the classification of Sjögren’s syndrome as reference methods. Seven of these studies [31,35-37,41,43,44] used the European Community Study Group on diagnostic criteria for Sjögren’s syndrome as reference method [48-52]. Two studies [32,34] used the Copenhagen Criteria for the classification of Sjögren’s syndrome [53] as reference method and two studies [33,45] used the proposed criteria for Sjögren’s syndrome according to Fox et al. [54] as a reference method. The cut-off values defining salivary gland hypofunction in the studies not using the above mentioned reference methods are presented in Table 4.

### Diagnostic accuracy

In all studies, the results of the test method were compared to those of a reference method [4,31-47]. These studies were interpreted according to the protocol based on the QUADAS tool [30].

**Figure 2:**
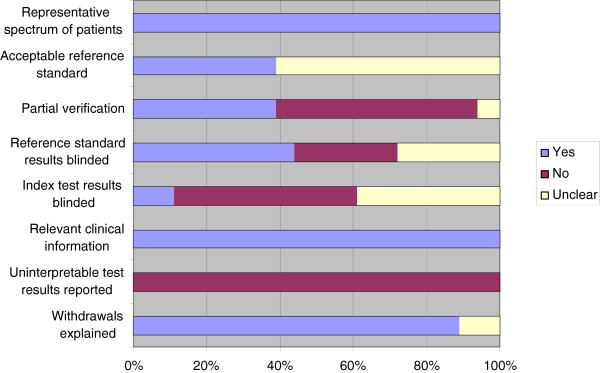
**Quality assessment. **Quality assessment of individual QUADAS items presented as percentages across all included studies.

Based on this, it can be reported that the patient selection criteria were not clearly described [38-40,47], and the test method [4] or the reference method [4,33,39,41,44] were not described in sufficient detail to allow the study to be reproduced. None of the included studies reported uninterpretable/intermediate results or data on observer or instrument variation. Authors’ judgements regarding seven of the quality items in the QUADAS tool (Table 2, questions no 1,4,6,9,10,11,12 and 13) are presented in Figure 2.

Seven of the included studies [4,31,37,41,44,45,47] presented their results as a percentage of correct diagnoses.

### Secretion tests

One study [31] examined the application of three different gel electrophoresis systems (SDS-PAGE, anionic-PAGE, immobilized pH gradient (IPG)) in the diagnosis of Sjögren’s syndrome. Tests of accuracy revealed that the immobilized pH grading system had a specificity of 97%, sensitivity of 95%, positive predictive value of 97% and negative predictive value of 95% in the diagnosis of Sjögren’s syndrome.

In the study by Kalk et al. [37], reference values of several salivary variables, i.e. sodium, chloride and phosphate concentration in stimulated parotid and submandibular/sublingual saliva, unstimulated and stimulated submandibular/sublingual flow rates and lag phase of parotid secretion were defined in order to diagnose Sjögren’s syndrome. The most accurate test, with a sensitivity of 85% and specificity of 96%, combined stimulated submandibular/sublingual flow rate and parotid sodium and chloride concentration.

Oral Schirmer’s test was used as test method in one study [41] for detecting salivary gland hypofunction. Cut-off values for the unstimulated test (≤ 30 mm/5 min, ≤ 40 mm/5 min, ≤ 50 mm/5 min, ≤ 60 mm/5 min), and for the stimulated test (≤ 50 mm/5 min, ≤ 60 mm/5 min, ≤ 70 mm/5 min, ≤ 80 mm/5 min) were examined and sensitivity, specificity, positive predictive values and negative predictive values were calculated. A cut-off value of ≤30 mm/5 min for the unstimulated sialometric test using Oral Shirmer’s test provided a sensitivity of 67.9% and a specificity of 62.8%. A cut-off value of ≤ 80 mm/5 min for the stimulated test showed a sensitivity of 87.6%, specificity of 67.5%, positive predictive value of 55.7% and a negative predictive value of 7.8%.

### Functional tests

A wafer test was used as a test method in one study [45] to predict early salivary gland dysfunction and xerostomia. Different cut-off values (3–7 min) were examined and the best balance between sensitivity and specificity was seen with a cut-off value of 4 min (wafer 4). This cut-off value exhibited a sensitivity of 92.9%, specificity of 71.7%, positive predictive value of 31.7%, negative predictive value of 98.6% and accuracy of 74.3%. With increasing time, the specificity for reduced salivary flow increased. Wafer 4 was compared with the European questionnaire [48] in order to validate the test as a screening instrument for low salivary flow and xerostomia. For low salivary flow, the European questionnaire [48] and the wafer 4 showed identical sensitivity. However, wafer 4 showed higher specificity, positive predictive value, negative predictive value, and accuracy. For xerostomia, wafer 4 showed higher sensitivity and negative predictive value when compared to the European questionnaire [48].

An indirect method (candy weight loss) was used as a test method in one study [47] to determine salivary output level. Significant associations were found when comparing candy weight loss with stimulated parotid and submandibular and sublingual saliva. When using a cut-off value of 0.23 g candy loss, the sensitivity was 92%, specificity 85% and the positive predictive value 82%.

### Questionnaires

Sreebny and Valdini [4] determined the validity of using symptoms related to dry mouth by using a questionnaire, to screen patients for salivary gland hypofunction. The study population was classified into three groups according to their ability to produce unstimulated whole saliva. Flow rates of ≤ 0.1 ml/min were characterised as “abnormal”, between 0.11 and 0.2 ml/min as “low normal”, and > 0.2 ml/min as “normal”. Determinations of sensitivity, specificity and negative and positive predictive values were made for xerostomia alone and for xerostomia in combination with three symptoms that were closely associated with it. A positive answer to the question “Does your mouth usually feel dry” showed a sensitivity of 93%, specificity of 68%, positive predictive value of 54% and negative predictive value of 98%. When an additional three symptoms were taken into account (difficulty with speech, trying to keep mouth moist and getting out of bed to drink), the specificity increased to 91% and the positive predictive value increased to 75%.

### Secretion/glandular morphology

A combination of tests for the classification of the oral component of Sjögren’s syndrome was examined in the study by Pennec et al. [44]. The tests examined were salivary flow rate (unstimulated whole saliva), salivary lysozyme, salivary lactoferrin, sialography, salivary gland scintigraphy and labial salivary gland biopsy. The authors concluded that the most efficient combination of test for the oral component of Sjögren’s syndrome was salivary gland biopsy (sensitivity 95%, specificity 75%, positive predictive value 90%, negative predictive value 14%) plus salivary flow rate (sensitivity 68%, specificity 81%, positive predictive value 90%, negative predictive value 50%) or salivary lactoferrin (sensitivity 58%, specificity 75%, positive predictive value 82%, negative predictive value 53%).

## Discussion

The aim of systematic reviews is to identify and evaluate all available research evidence relating to a particular objective. An essential part of any systematic review is the quality assessment of individual studies. Aspects such as study design, methods of sample recruitment, the execution of the tests and the completeness of the study report relate to the overall quality. The results of a systematic review will be biased if the results of individual studies are synthesised without any consideration of quality in terms of potential for bias, lack of applicability and the quality of reporting.

QUADAS [30] was the first systematically developed, evidence based quality assessment tool to be used in systematic reviews of diagnostic accuracy studies. The QUADAS tool [30] contains a detailed explanation of the intention of each item, situations when the item does not apply and how to score items. This allows for minor adaptations in specific areas. The QUADAS tool [30] does not incorporate quality scores to assess the level of evidence. Since the importance of individual items and their potential biases may vary according to the context in which they are applied, incorporation of quality scores into the results of a review may generate different magnitudes of bias and lead to different conclusions regarding the effect of study quality on estimates of diagnostic accuracy. Instead, it has been proposed that a systematic review should involve a component approach, where the association of individual quality items with test accuracy are investigated individually [55].

The QUADAS tool [30] was used in the present systematic review since it is a standardised approach to quality assessment and since the criteria needed to assess the quality of diagnostic test evaluations differ from those needed to assess evaluations of therapeutic interventions [56].

In the present review, modifications were done to adapt the QUADAS tool [30] to better correspond to the objectives of the study. Two questions (numbers 6 and 7) were excluded from the 14 questions of the final QUADAS tool [30] and two were added from the original list of 28. In addition, question number 3 was modified into two sub-questions.

When interpreting the studies with the aid of the QUADAS tool [30] it could be established that some studies showed shortcomings in describing the selection criteria clearly and in describing the test method or the reference method in such detail that it could be reproduced. A sufficient description of the tests is important since variations in measures of diagnostic accuracy can be traced back to differences in the execution of the tests. A clear description is also needed in order to implement the test in another setting. None of the included studies reported uninterpretable/intermediate results or data on observer or instrument variation. A diagnostic test can produce uninterpretable/intermediate results with varying frequency. If these results are removed from the analysis, it may lead to biased assessments of the test characteristics. Furthermore, it can be questioned if some of the studies presented appropriate results.

When scoring QUADAS items [30] as unclear it is difficult to be certain if this indicates poor methods with the attendant consequences for bias and/or variation, or simply poor reporting of a methodologically sound study. The STARD initiative [57] has proposed standards for the reporting of diagnostic accuracy studies. If these standards are widely adopted, reviewers might be able to assess methodological quality rather than the quality of reporting. The aim of test accuracy studies is to assess how well a test can distinguish between subjects with and without the disease/condition of interest. There are two basic types of test accuracy study: (1) The single-gate design which includes participants in whom the disease status is unknown, and compares the results of the index test with those of a reference standard used to confirm diagnosis. This design is broadly representative of the setting in which the test would be used in practice. (2) The two-gate design compares the results of the index test in patients with an established diagnosis of the target condition with its results in healthy controls or controls with another diagnosis. This design has inherent problems that may lead to bias. The inclusion of healthy controls is likely to lead to over estimations of specificity and the selective inclusion of cases with more advanced disease is likely to lead to over estimations of sensitivity [58]. The two-gate studies can however be useful in the earlier phases of test development.

Addressing the question formulated to specify the problem, it can be concluded that whilst a variety of tests to diagnose oral dryness have been examined, only a few have been validated in terms of diagnostic accuracy. Eight of the included studies presented their results as percentage of correct diagnoses. Four of these studies used European Community Study Group on classification criteria for Sjögren’s syndrome [48-52] as reference method. The European classification criteria for Sjögren’s syndrome were developed and validated between 1989 and 1996 and have received broad acceptance by the scientific community. Since the reference standard is an important determinant of the diagnostic accuracy of a test, it raises the question of why all of studies aimed at the evaluation of tests for the diagnosis of Sjögren’s syndrome did not use the same reference method.

Although these criteria have received a broad acceptance, some criticism has been raised concerning the inclusion of subjective test (symptoms), physiologic measures that lack specificity and alternate objective tests that are not diagnostically equivalent.

Recently, the American College of Rheumatology [59] proposed new classification criteria for Sjögren’s syndrome. These criteria are based on expert opinion elicited using the nominal group technique and analyses of data from the Sjögren’s International Collaborative Clinical Alliance [60]. The proposed criteria are: 1) positive serum anti-SSA and/or anti-SSB or (positive rheumatoid factor and antinuclear antibody titer ≥1:320), 2) ocular staining score ≥3, or 3) presence of focal lymphocytic sialadenitis with a focus score ≥1 focus/4 mm^2^ in salivary gland biopsy samples. Case definition requires at least 2 of the 3 above mentioned objective features. Thus, only objective tests and not subjective tests (symptoms) are included since symptoms of dry mouth and/or eyes can lead to misclassification of asymptomatic patients. For the salivary and ocular phenotypic features of Sjögren’s syndrome the results did not identify any suitable alternate tests besides labial salivary gland biopsy. While unstimulated salivary flow rate <0.1 ml/min had good sensitivity, it had low specificity compared to the labial salivary gland biopsy to measure focal lymphocytic sialadenitis with a focus score ≥1 [59]. Seven of the studies interpreted in this review evaluated different tests for determining decreased salivary flow and used sialometry as a reference method. These studies revealed heterogeneity with respect to source of secretion whether unstimulated or stimulated. Cut-off values defining salivary gland hypofunction also varied. As stated earlier, without proper individual baseline information, it is almost impossible to ascertain if the level of a patient’s salivary flow rate is below the ‘normal’. When using sialometry for diagnosing salivary dysfunction it can be argued if the method is used as a diagnostic tool or rather as a verification of an already established condition. Sreebny [61] proposed that the low cut-off values should be viewed as values which “flag” or “raise suspicion” about the presence of a disease. They do not indicate that the person who demonstrates such values definitely has a disease.

The fact that there is no global consensus regarding the terminology of oral dryness, although many authors distinguish between xerostomia, denoting the subjective feeling, and hyposalivation, denoting a decreased salivary flow rate, creates a problem for research, diagnosis, and therapy. As for research, this problem is illustrated when using Medical Subject Headings (MeSH). MeSH is the National Library of Medicine’s controlled vocabulary thesaurus used for indexing articles for PubMed. The MeSH database defines xerostomia as decreased salivary flow, which is incorrect since a sensation of oral dryness can occur in subjects with a normal salivary flow. Nederfors [12] proposed to divide the term “salivary gland hypofunction” into 3 different entities: xerostomia, denoting the subjective feeling; hyposalivation, denoting the decreased salivary flow rate; and altered saliva composition. This classification accepts that xerostomia may exist without signs of hyposalivation, that hyposalivation may be a symptomless condition and that an altered saliva composition may exist even if the saliva secretion rate is unaffected and without subjective symptoms. These three entities are inter-related and can influence each other in different ways.

Over the last decade, advances have been made regarding proteomic and genomic approaches to identify potential biomarkers that may be used in the detection of different diseases, e.g. Sjögren’s syndrome [62]. Saliva is a biofluid that is readily accessible via noninvasive methods, and therefore a perfect medium to be explored for purposes to monitor health status, disease onset and progression, and treatment outcome. Salivary diagnostic technologies identifying specific biomarkers associated with disease may in the future be used to verify general diseases behind salivary gland hypofunction [63]. It should also be mentioned that in the absence of an efficient treatment, a diagnostic method has little value. The basic causes of oral dryness are difficult to treat and many methods have been tested to stimulate saliva secretion and ease the patient’s discomfort, e.g., saliva-stimulating tablets and artificial saliva. Several studies have evaluated the efficacy of such preparations but there is no documented evidence of their effect on oral health [64-66].

Currently, diagnostic methods are addressing quantity and content of saliva in bulk and few qualitative tests of saliva, in bulk or of saliva as an adsorbed thin film, are at this date available for describing the protective functions of saliva.

Since changes in the protective functions of saliva may occur, there is a need for effective diagnostic criteria and functional tests in order to discern which individuals with oral dryness will require oral treatment, such as alleviation of discomfort and/or prevention of diseases.

An important component in determining the usefulness of a test is the evaluation of the diagnostic accuracy, but the clinical value lies in improving a patient’s condition or health. The clinical value, i.e. how the results of a test affects the clinical decision-making and the effect on the patient’s wellbeing are important factors when evaluating diagnostic tests or methods. A method with high diagnostic accuracy may not always be efficient and useful for the patient. Studies that investigate the value of diagnostic interventions are scarce and seldom available for new test methods. In addition, appropriate reference standards for many disorders are lacking.

## Conclusions

The aim of this systematic review was to evaluate the quality of evidence for diagnostic methods used to identify oral dryness and their clinical application.

After assessing the quality of the retrieved studies, it may be concluded that the evidence for the efficacy of clinical methods to assess oral dryness is sparse. When evaluating the retrieved studies by using the QUADAS tool [30], many of the studies exhibited shortcomings. Standards for the reporting of diagnostic accuracy studies have been suggested, such as the STARD initiative [30]. If these standards are widely adopted, the quality of reporting will be improved and the methodological quality of diagnostic accuracy studies will be easier to assess. This will, as a consequence, benefit patients.

A global consensus regarding the terminology of oral dryness is needed in order to facilitate diagnosis and treatment and continued research.

Changes in the protective functions of saliva may occur, which might affect oral health.

There is a need for effective diagnostic criteria and functional tests in order to detect those individuals with oral dryness who may require treatment, such as alleviation of discomfort and/or prevention of diseases.

## Competing interests

The authors declare that they have no competing interests.

## Authors’ contributions

CDL and CC conceived the study. CDL, CW, MS and CC designed the study and CDL was responsible for the literature search. PTL was responsible for the search strategy. CDL, CW, MS and CC appraised the identified publications. CDL drafted the manuscript. All authors read and approved the final manuscript.

## Pre-publication history

The pre-publication history for this paper can be accessed here:

http://www.biomedcentral.com/1472-6831/12/29/prepub

## Supplementary Material

Additional file 1: Table S1 The protocol used in the assessment against the stated inclusion criteria. Click here for file
