# Supplementary material for: A systematic review of methods to diagnose oral dryness and salivary gland function
Source: BMC Oral Health. 2012 Aug 8;12:29. doi: 10.1186/1472-6831-12-29 (PMC3573918; doi:10.1186/1472-6831-12-29)
Supplement: Additional file 1: Table S1 — The protocol used in the assessment against the stated inclusion criteria. [file 1472-6831-12-29-S1.doc]

Additional file 1: Table S2 - **Protocol**

The protocol used in the assessment against the stated inclusion criteria.

Title……………………………………………………………………………………………

Author(s)………………………………………………………………………………………

Journal…………………………………………… Year……… Volume………..

Pages……………………

|  | Yes | No | Uncertain | Comments |
| --- | --- | --- | --- | --- |
| Publication in English |  |  |  |  |
| Study conducted on humans |  |  |  |  |
| Oral dryness primary condition |  |  |  |  |
| Study population presenting symptoms  and/or findings of oral dryness |  |  |  |  |
| Standardized conditions for donors |  |  |  |  |
| Control group |  |  |  |  |
| Description of study design |  |  |  |  |
| Description of saliva collection procedures |  |  |  |  |
| Description of the patient population that was tested |  |  |  |  |
| Ethical approval |  |  |  |  |
| Description of statistical analysis |  |  |  |  |

Will the study be included in the systematic review? Yes No
